# Inflammatory breast cancer associated with amyopathic dermatomyositis: a case report

**DOI:** 10.1186/s40792-020-01066-7

**Published:** 2020-11-11

**Authors:** Gaku Inaguma, Akihiko Shimada, Junya Tsunoda, Tomohiko Matsuzaki, Tomohiko Nishi, Hiroaki Seki, Hidetoshi Matsumoto

**Affiliations:** Department of Surgery, Keiyu Hospital, 3-7-3 Minatomirai, Nishi-ku, Yokohama, Kanagawa 220-8521 Japan

**Keywords:** Inflammatory breast cancer, Neoadjuvant chemotherapy, Amyopathic dermatomyositis, Paraneoplastic syndrome

## Abstract

**Background:**

Dermatomyositis is associated with malignant tumors including breast cancer, and inflammatory breast cancer is considered to have a poorer prognosis than most breast cancers.

**Case presentation:**

A 74-year-old Asian woman, developed erythema on her face, back, and the back of her hands, 3 weeks before attending our department. At the same time, she had noticed a right breast mass and redness of the skin of the breast. The clinical findings and vacuum aspiration biopsy diagnosed inflammatory breast cancer and neoadjuvant chemotherapy was performed. The mass and enlarged axillary lymph nodes had shrunk, therefore a total mastectomy was performed. The sentinel lymph node biopsy was negative. She was discharged 7 days after surgery without any complications. She has received a postoperative aromatase inhibitor and is alive without recurrence. The dermatomyositis also began to improve with the start of her chemotherapy and has not recurred since the surgery.

**Conclusions:**

Neoadjuvant chemotherapy was performed for inflammatory breast cancer with dermatomyositis, and tumor shrinkage was confirmed. A total mastectomy without axillary lymph node dissection was performed. Dermatomyositis and breast cancer have not recurred. Dermatomyositis may have been a paraneoplastic syndrome due to breast cancer.

## Background

Dermatomyositis is commonly associated with malignant tumors including breast cancer. It is known that dermatomyositis can occur with nasogastric cancer in Asians [1]. Although the majority of cases are idiopathic, in approximately 15–30% of cases of adult-onset dermatomyositis, it develops as a paraneoplastic syndrome caused by an underlying malignancy [2]. Paraneoplastic syndromes are considered to be caused by cancer that are based on the immune response to the tumor or on humoral factors secreted by the tumor.

Amyopathic dermatomyositis has classic cutaneous findings of dermatomyositis, but lacks clinical evidence of muscle weakness [3]. Amyopathic dermatomyositis accounts for less than 20% of all dermatomyositis cases and is often associated with breast cancer [4].

Inflammatory breast cancer accounts for about 2.5% of all breast cancers [5], and has a poor prognosis, with a 5-year survival rate of less than 30% even with multidisciplinary treatment [6].

## Case presentation

**Fig. 1:**
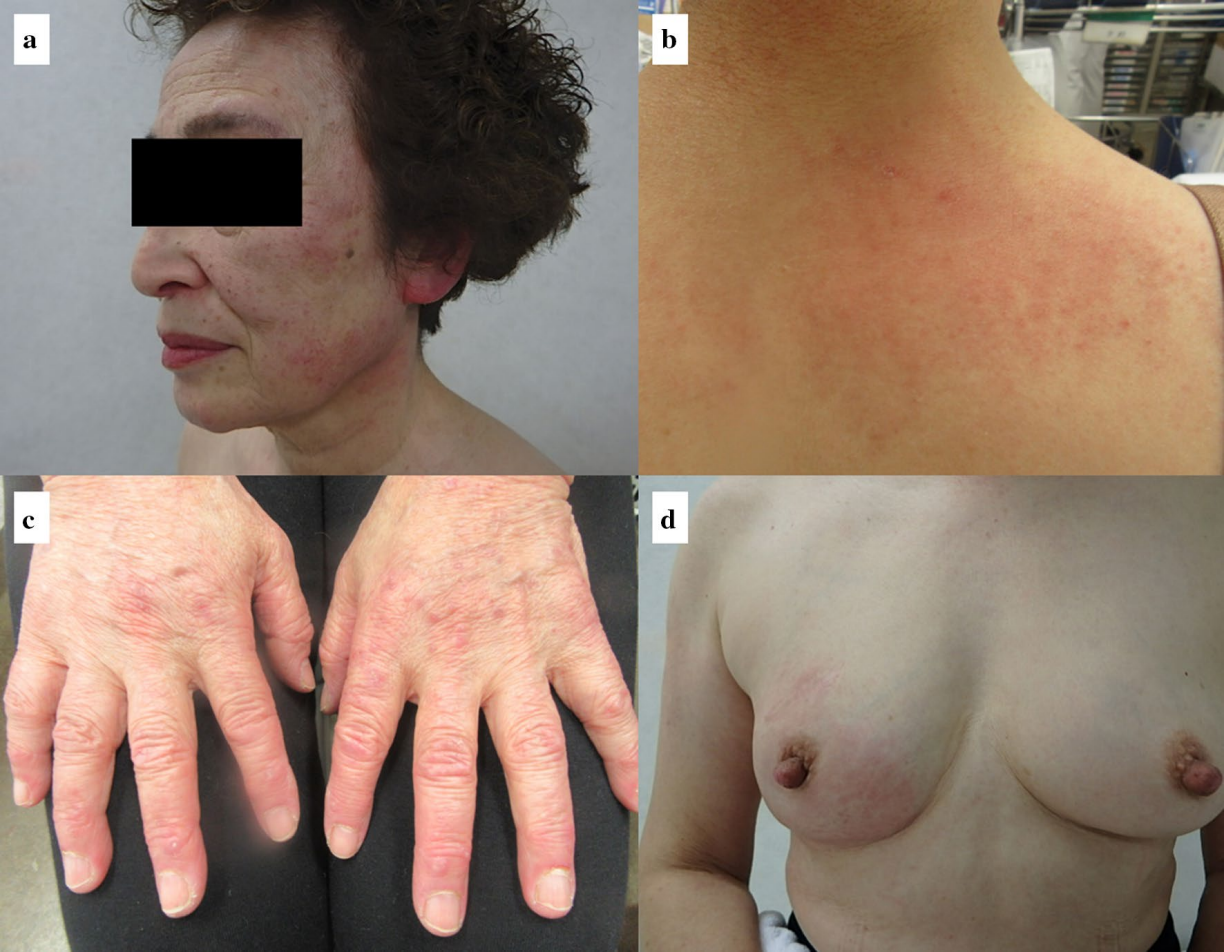
**a** Redness was observed on the forehead, cheeks, and neck. **b** Redness was observed on the upper back—the ‘shawl sign’. **c** Multiple hyperkeratotic and erythematous papules were observed over the metacarpophalangeal and interphalangeal joints of both hands. **d** Redness of the skin of the right breast was observed

**Fig. 2:**
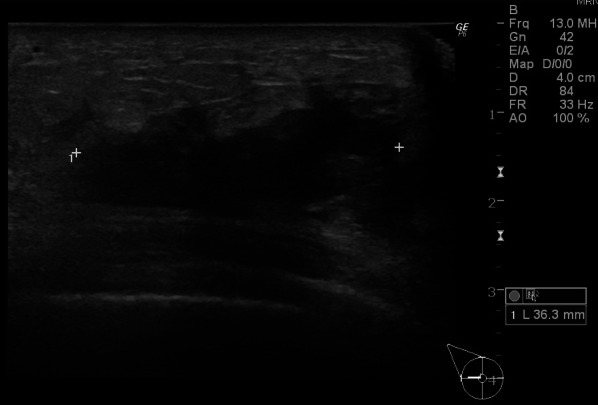
Breast ultrasonography revealed a 36-mm irregular mass at the 9 o’clock position

**Fig. 3:**
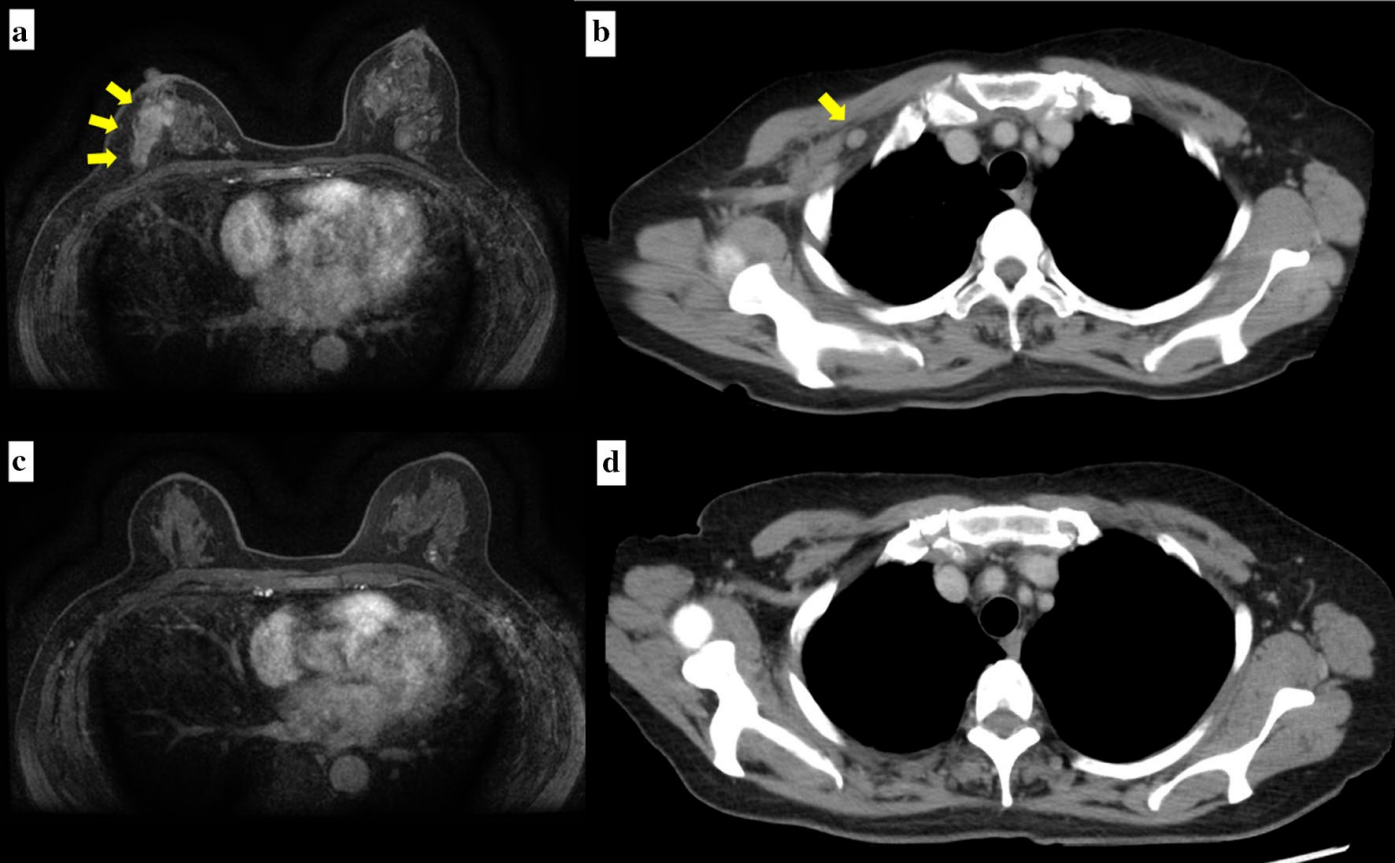
**a** MRI (T2-weighted image) showed a high-signal mass in the right breast before chemotherapy. **b** CT showed a swollen axillar lymph node before chemotherapy. **c** MRI (T2-weighted image) showed that the mass had disappeared after chemotherapy. **d** CT showed that the lymph node had disappeared after chemotherapy

A 74-year-old Asian woman with no family history of significant medical problems presented with erythema on her face, back, and the back of her hands which had developed 3 weeks earlier (Fig. 1a–c). At the same time, she noticed a right breast mass and redness of the skin of the breast (Fig. 1d). Breast ultrasonography revealed a hyperechoic view of the entire skin of the right breast and a 36-mm irregular mass at the 9 o’clock position (Fig. 2). A T2-weighted magnetic resonance imaging (MRI) examination showed a high-signal mass at the same area, and enlargement of the right axillary lymph nodes (Fig. 3a, b).

**Fig. 4:**
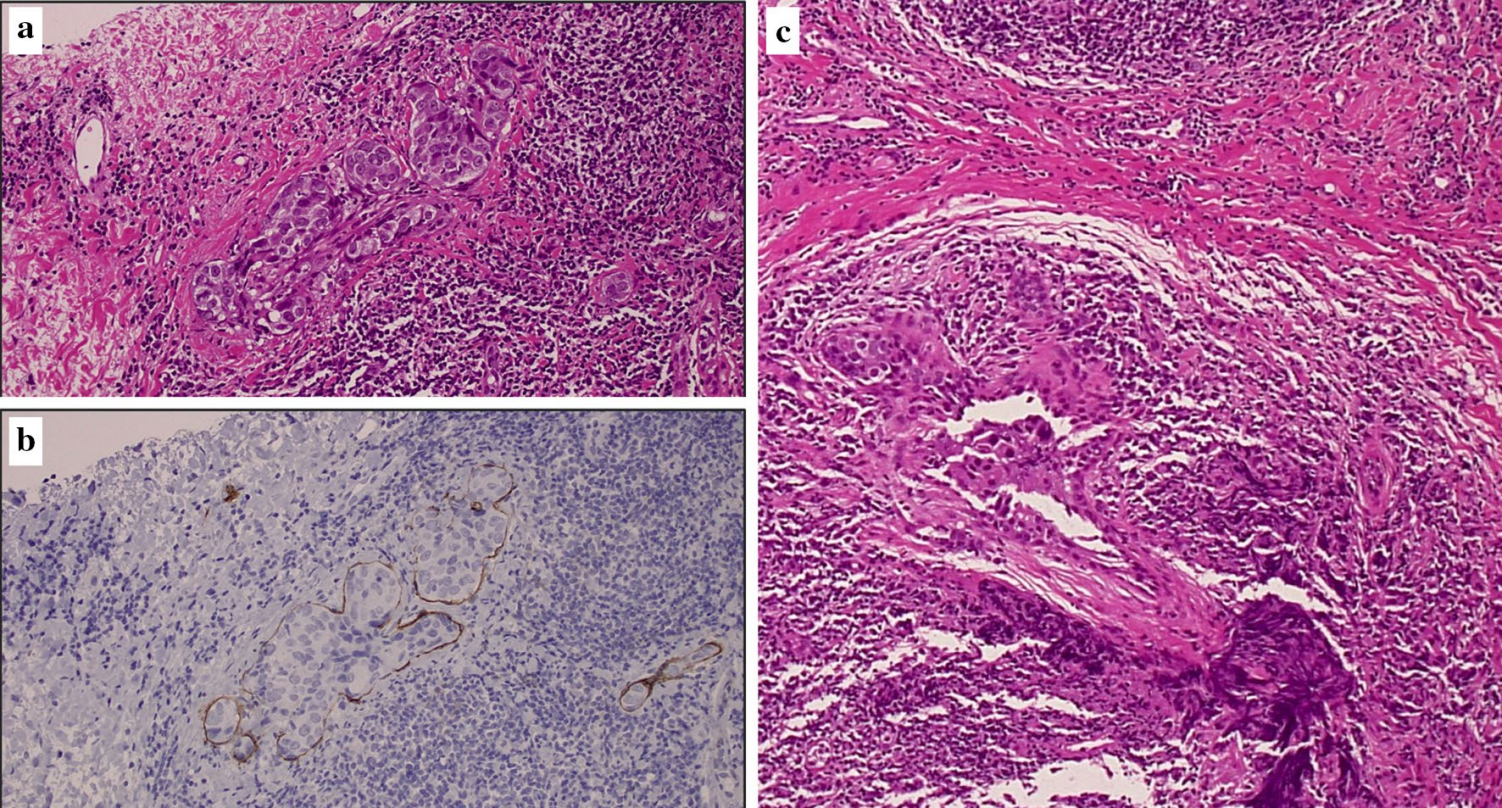
Histopathological examinations of biopsy before chemotherapy. **a** Atypical cells with enlarged chromatin nuclei and eosinophilic endoplasmic reticulum form vesicular nests on hematoxylin–eosin stain. **b** D2-40 staining is positive, indicating that the tumor cells have infiltrated the lymphatic vessels. **c** Atypical cells with enlarged nuclei and eosinophilic sporangium form a vesicular structure and proliferate. It is a highly atypical invasive ductal carcinoma of the breast

A vacuum aspiration biopsy was performed. It showed that atypical cells with enlarged chromatin nuclei and eosinophilic endoplasmic reticulum form vesicular nests on hematoxylin–eosin stain. We made a diagnosis of invasive ductal carcinoma that was suspected to be inflammatory breast cancer (Fig. 4a–c). The tumor was positive for both estrogen (about 100%) and progesterone (over 90%) receptors, HER2 was negative, and the Ki-67 index was 40%. A computed tomography (CT) scan revealed no metastases, and the stage was IIIC (cT4dN3aM0) in TNM classification.

The patient was also referred to a dermatologist. The shawl sign was seen on her upper back (Fig. 1b). Multiple hyperkeratotic and erythematous papules were observed over the metacarpophalangeal and interphalangeal joints of both hands (Gottron papule, Fig. 1c). The patient did not have muscle weakness. Blood tests showed that her creatine kinase, aspartate aminotransferase, and aldolase were normal, the anti-transcriptional intermediary factor 1-γ antibody (specific to amyopathic dermatomyositis) was positive, and the test for anti-aminoacyl tRNA synthetase antibody was negative.

Based on these findings, she was diagnosed with amyopathic dermatomyositis, and prednisolone was started at 20 mg/day. She also received 4 cycles of epirubicin–cyclophosphamide therapy. The total dose of epirubicin was 425 mg and cyclophosphamide was 2970 mg. Subsequent ultrasonography and MRI revealed that the tumor had shrunk remarkably, the swollen right axillary lymph node had disappeared (Fig. 3c, d) as had the rash on the right breast. Therefore, a total mastectomy and sentinel lymph node biopsy were performed. No tumor cells were found in the sentinel lymph nodes and axillary lymph node dissection was not performed.

Breast pathology showed only non-invasive carcinoma in the duct. The sentinel lymph nodes in the permanent specimen showed fibrosis. Chemotherapeutic effect was Grade 2b in the General Rules for Clinical and Pathological Recording of Breast Cancer (18^th^ edition) [7]. Therefore, postoperative stage was ypTisN0M0.

The dermatomyositis began to improve during chemotherapy. The prednisolone was reduced to 5 mg/day after surgery, without any worsening of her symptoms. No postoperative chemotherapy was performed, and she has been taking letrozole (an aromatase inhibitor) for a year and has not seen a recurrence.

## Discussion

Dermatomyositis is commonly associated with malignant tumor. Specific antibody (anti-transcriptional intermediary factor 1-γ antibody) that increase the risk of cancer have been found in about half of dermatomyositis patients. It has been confirmed in this case [8]. However, the mechanism of cancer development is unknown.

It has been reported that breast cancer that is not inflammatory is associated with dermatomyositis and that treatment of the breast cancer improves the symptoms of dermatomyositis, which is a feature of the paraneoplastic syndrome [9]. In this case, oral prednisolone was started concurrently with the neoadjuvant chemotherapy. The improvement of the symptoms of dermatomyositis before surgery was thought to be due to the oral prednisolone. However, when the dose of prednisolone was reduced after surgery, the symptoms of dermatomyositis did not worsen, therefore it may have been due to the paraneoplastic syndrome.

Neurological disease has been reported as another manifestation of the paraneoplastic syndrome caused by breast cancer. Murphy et al. retrospectively analyzed cases of paraneoplastic neurologic syndrome caused by breast cancer and reported that 57% of patients developed neurologic symptoms before their breast cancer diagnosis [10]. In some cases, treatment of the breast cancer has been reported to improve the neurological symptoms, similar to the response in this case [11].

It has been found that regenerating cells of myositis muscle express high levels of myositis-specific self-antigens, but these antigens are rarely expressed in normal muscle cells [12]. Some of these antigens are often expressed in breast and lung cancers and are thought to cause symptoms of muscle weakness when muscle tissue with specific self-antigens is impaired in a cross-reaction by the immune response to the tumor. This mechanism can be described as a paraneoplastic syndrome, but it can only be established if myositis and tumor are present at the same time. Whether the tumor preceded the myositis or not cannot be explained by this theory.

## Conclusion

We performed neoadjuvant chemotherapy for inflammatory breast cancer with dermatomyositis and confirmed that the tumor had shrunk, so we performed a total mastectomy without axillary lymph node dissection. Dermatomyositis may have been a sign of the paraneoplastic syndrome due to breast cancer.

## Data Availability

Data sharing is not applicable to this article, as no datasets were generated or analyzed during the current study.

## Abbreviations

MRI: Magnetic resonance imaging
CT: Computed tomography
